# Supplementary material for: AKT-dependent signaling of extracellular cues through telomeres impact on tumorigenesis
Source: PLoS Genet. 2021 Mar 9;17(3):e1009410. doi: 10.1371/journal.pgen.1009410 (PMC7942993; doi:10.1371/journal.pgen.1009410)
Supplement: S1 Table — (DOC) [file pgen.1009410.s001.doc]

**S1 Table:** Primers used in this study

| **Primer** | **Sequence (5´-3´)** |
| --- | --- |
| TRF1273A-F | ACAAGAACAATAGCATCTCAAGATAAACCT |
| TRF1273A-R | AGGTTTATCTTGAGATGCTATTGTTCTTGT |
| TRF1344A-F | AGAAGAGTAGGAGCACCTCAAAGTACAAAA |
| TRF1344A-R | TTTTGTACTTTGAGGTGCTCCTACTCTTCT |
| TRF1358A-F | AGCAGAAGAGCCGCAGAAAGCAGAATACCT |
| TRF1358A-R | AGGTATTCTGCTTTCTGCGGCTCTTCTGCT |
| TRF1273D-F | ACAAGAACAATAGATTCTCAAGATAAACCT |
| TRF1273D-R | AGGTTTATCTTGAGAATCTATTGTTCTTGT |
| TRF1358D-F | AGCAGAAGAGCCGATGAAAGCAGAATACCT |
| TRF1358D-R | AGGTATTCTGCTTTCATCGGCTCTTCTGCT |
| Seq-TRF1 | AATTACACCCTTGGAATCAG |
| RT-TRF1-F | TTCTAATGAAGGCAGCGGCA |
| RT-TRF1-R | GTTGCTGGGTTCCATGTTGC |
| RT-Tubulin-F | AGTGAAAACAATCTAACCAGAAA |
| RT-Tubulin-R | GGCCCGTGAAGATATG |
| sgRNA-T273A-F | CACCgACTTCTCAAGATAAACCTAG |
| sgRNA-T273A-R | AAACCTAGGTTTATCTTGAGAAGTc |
| sgRNA-T358A-F | CACCgAACAGGTATTCTGCTTTCAG |
| sgRNA-T358A-R | AAACCTGAAAGCAGAATACCTGTTc |
| g-T273A-F | GACCACCTTCGTAACGCTGA |
| g-T273A-R | CCCCAAGAATGGTAAACTATCCA |
| g-T358A-F | ACTCAAAACACTCCCCCTCT |
| g-T358A-R | ATGTCAGTACCAACACCCAACT |
| ss-DNA-T273A | AAAGTAGTAGAAAGCAAAAGGACAAGAACGATCGCTTCTCAAGATAAACCTAGTGGTAATGATGTTGAAATGGAAACTGAAGCTAATTTGGATACAAGAA |
| ss-DNA-T358A | AAAAAAGAAAGCAGAAGAGCTGCAGAAAGCAGAATACCTGTTTCAAAGAGTCAGCCAGTAACTCCTGAAAAACATCGAGCTAGAAAAAGACAGGTATTTGGT |
