# Supplementary figures and images for: AKT-dependent signaling of extracellular cues through telomeres impact on tumorigenesis
Source: PLoS Genet. 2021 Mar 9;17(3):e1009410. doi: 10.1371/journal.pgen.1009410 (PMC7942993; doi:10.1371/journal.pgen.1009410)

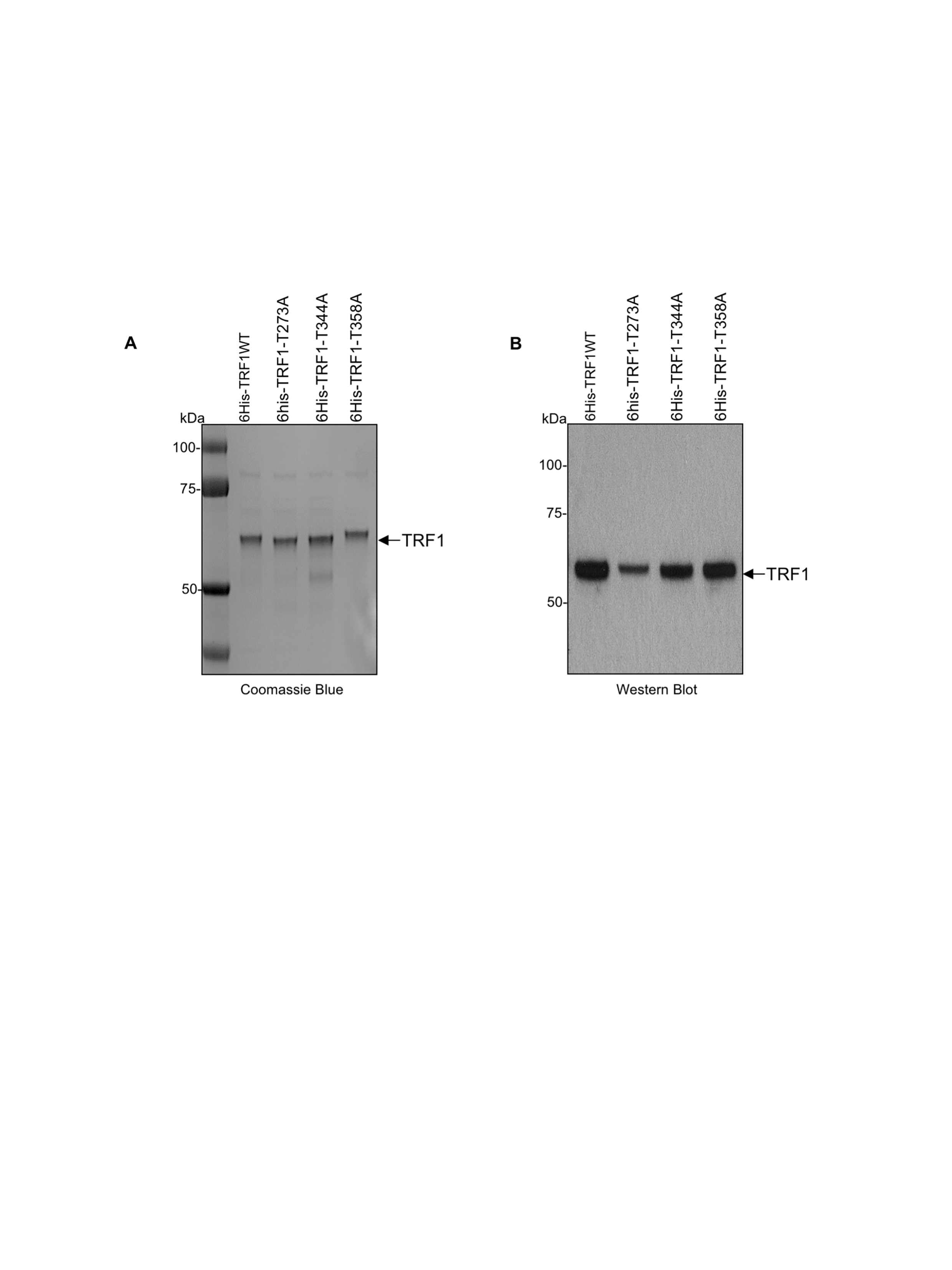

Supplement: S1 Fig — A-B. Coomassie stained SDS-PAGE gel (A) and western blot (B) of affinity purified His-TRF1-WT, His-TRF1-T273A, His-TRF1-T344A and His-TRF1-T358A (2μg). The MW ladder is shown to the left. (TIFF) [file pgen.1009410.s002.tiff]

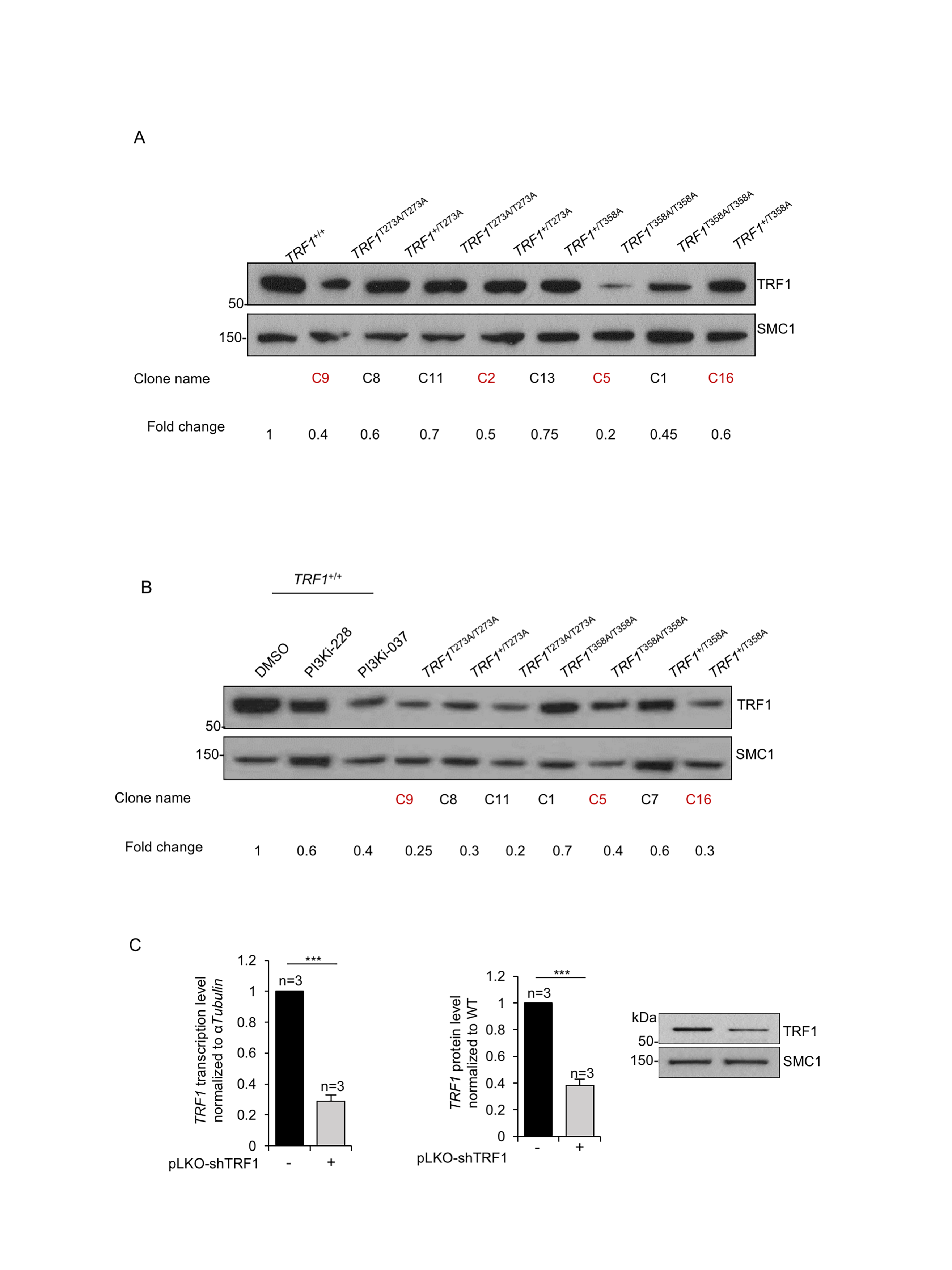

Supplement: S2 Fig — A-B. Representative western blot images of total nuclear TRF1 protein levels in wild type and in different independent heterozygous and homozygous clones. The heterozygous mutant TRF1+/T273A and TRF1+/358A clones correspond to C2 & C8 and to C7 & C13 & C16, respectively. The homozygous TRF1T273A/T273A and TRF1T358AT/358A mutant clones correspond to C9 & C11 and to C5 & C1, respectively. Those clones used throughout the manuscript are labeled in red. The fold change in TRF1 levels with regards to wild type cells is indicated below the images. SMC1 was used as a loading control. C. Quantification of TRF1 transcriptional levels by q-PCR and TRF1 protein levels in TRF1+/+ cells transfected with an sh-TRF1. SMC1 was used as a loading control. A representative western blot images of total nuclear TRF1 protein levels is shown in right panel. Error bars represent standard deviation. n number of independent experiments. Student’s t test was used for statistical analysis, P values are shown. ***, p ≤ 0.001. (TIFF) [file pgen.1009410.s003.tiff]

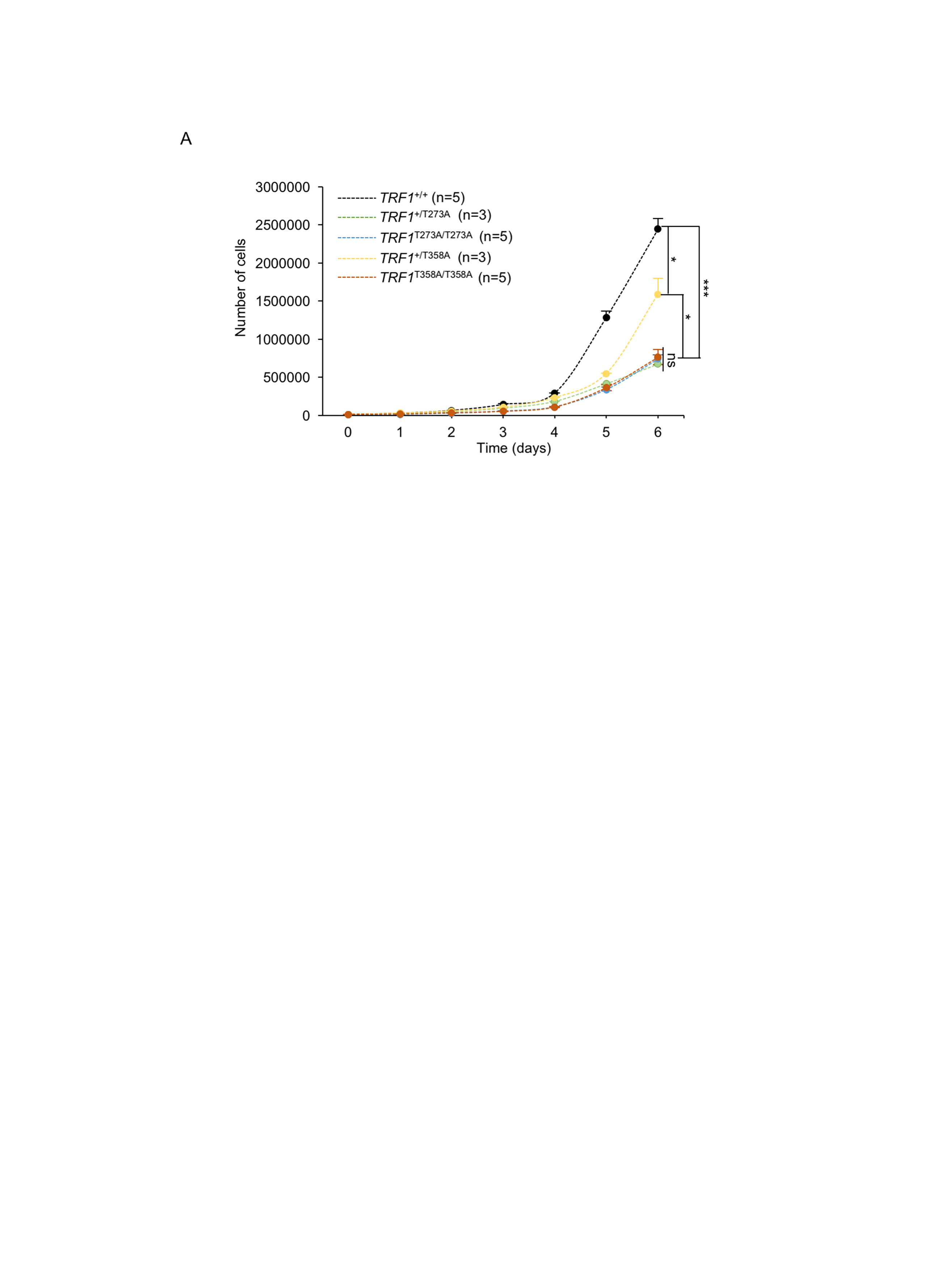

Supplement: S4 Fig — A. Growth rate of TRF1+/+, TRF1+/T273A, TRF1T273A/T273A, TRF1+/T358A and TRF1T358A/T358A cell lines. Student’s t test was used for statistical analysis, p ≤ 0.05; **, p ≤ 0.01; ***, p ≤ 0.001. Error bars represent ± SE. n number of independent experiments. (TIFF) [file pgen.1009410.s005.tiff]

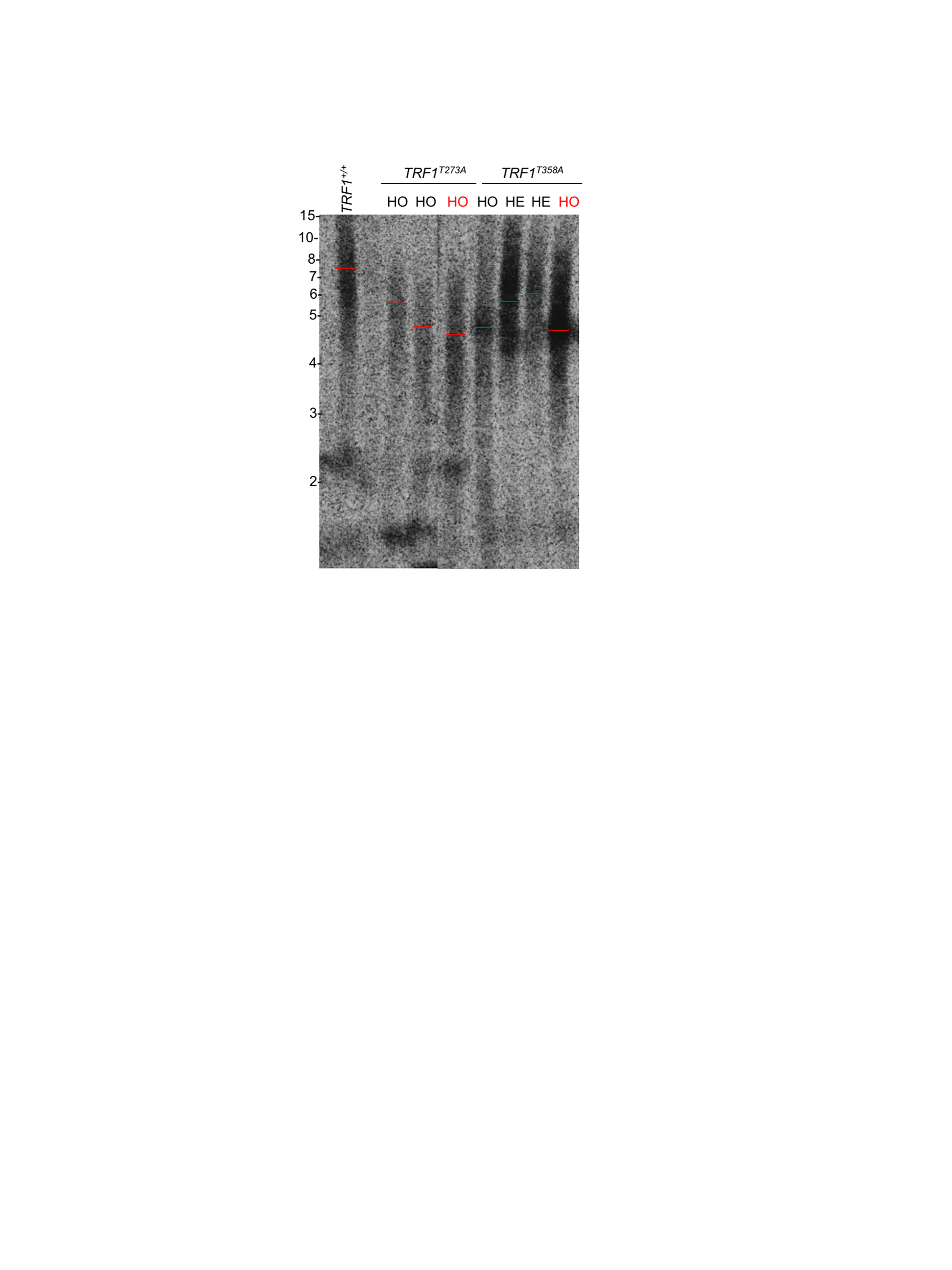

Supplement: S5 Fig — Telomeric restriction fragment (TRF) blot of TRF1T273A and TRF1T273A independent knock-in clones. HE and HO refer to heterozygous and homozygous clones, respectively. Numbers refer to molecular weight standards in Kb. Those clones used throughout the manuscript are labeled in red. (TIFF) [file pgen.1009410.s006.tiff]

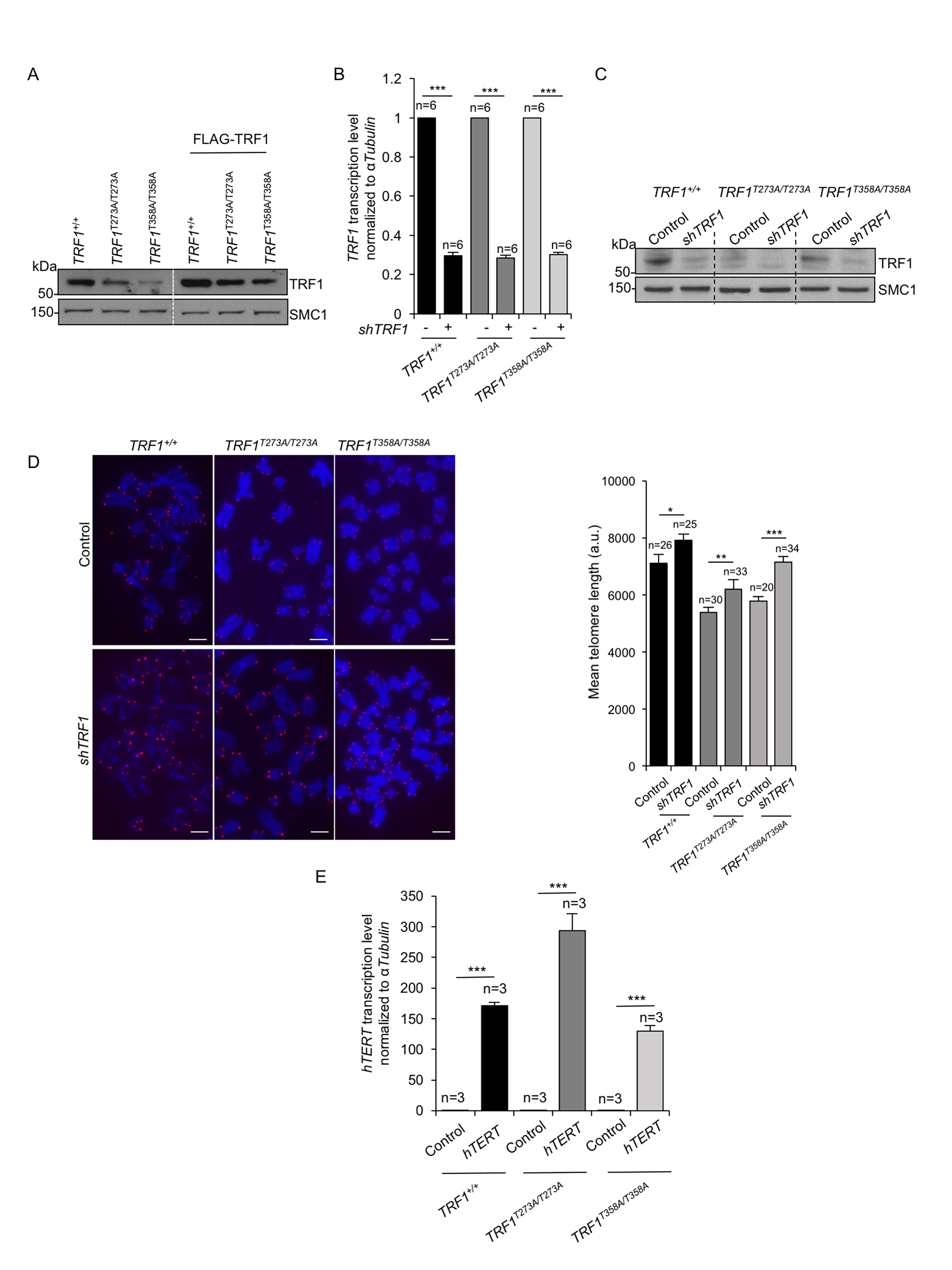

Supplement: S6 Fig — A. Representative western blot images of total nuclear TRF1 protein levels in TRF1+/+, TRF1T273A/T273A and TRF1T358A/T358A transfected with FLAG-TRF1. B. Quantification of TRF1 transcriptional levels by q-PCR in TRF1+/+, TRF1T273A/T273A and TRF1T358A/T358A cells transfected with an sh-TRF1. C. Representative western blot images of total nuclear TRF1 protein levels in TRF1+/+, TRF1T273A/T273A and TRF1T358A/T358A transfected with an sh-TRF1. D. Representative Q-FISH images of metaphases spreads and mean telomere length quantification from TRF1+/+, TRF1T273A/T273A and TRF1T358A/T358A cell lines transfected with an sh-TRF1 at passage 14. Scale bars, 5μm. Student’s t test was used for statistical analysis, p ≤ 0.05; **, p ≤ 0.01; ***, p ≤ 0.001. Error bars represent ± SE. n number of metaphases. E. Quantification of TERT transcriptional levels by q-PCR in TRF1+/+, TRF1T273A/T273A and TRF1T358A/T358A cells transfected pBABE-TERT. Student’s t test was used for statistical analysis, p ≤ 0.05; **, p ≤ 0.01; ***, p ≤ 0.001. Error bars represent ± SE. n number of independent experiments. (TIFF) [file pgen.1009410.s007.tiff]

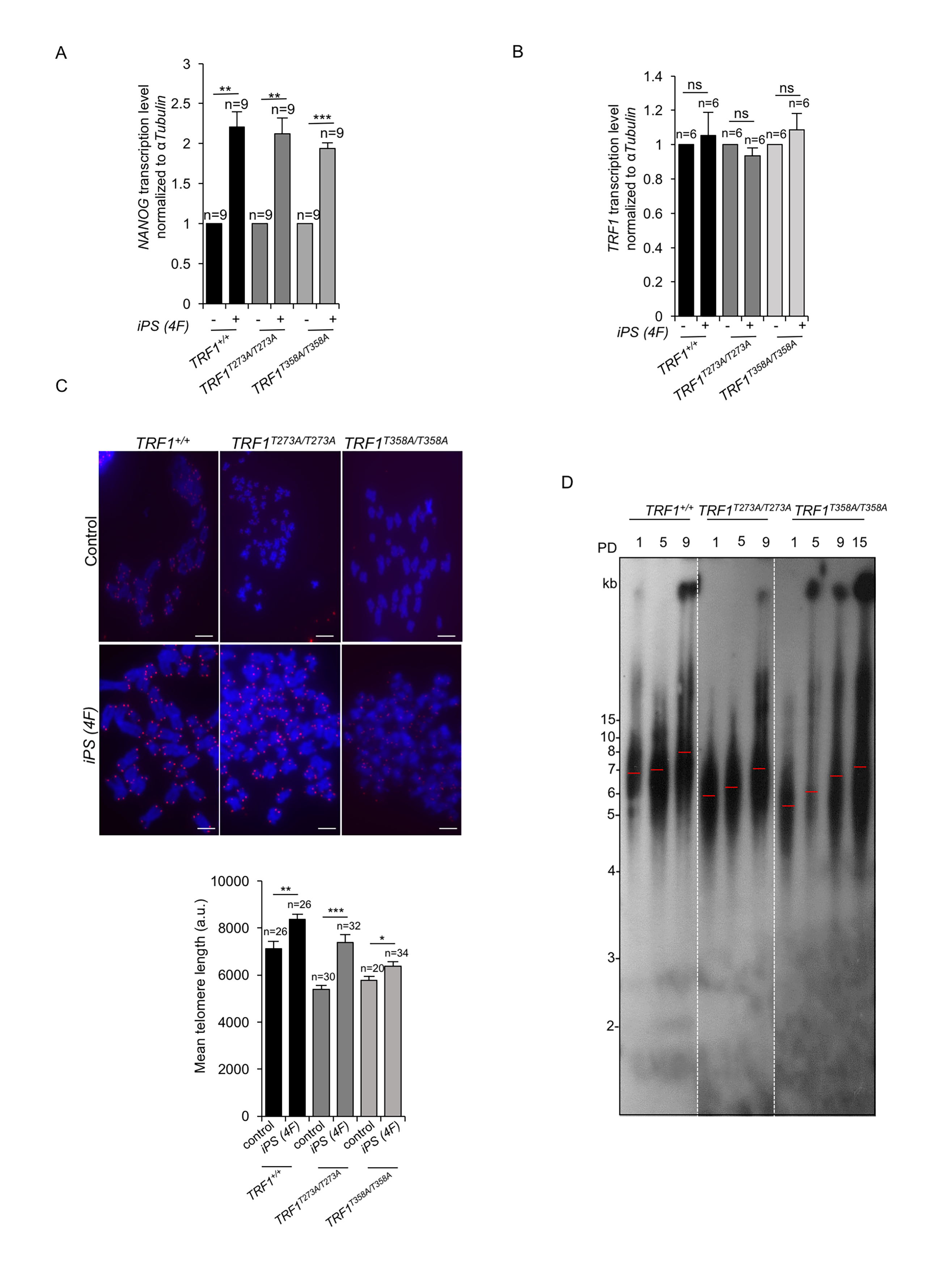

Supplement: S7 Fig — Quantification of NANOG transcriptional levels by q-PCR in TRF1+/+, TRF1T273A/T273A and TRF1T358A/T358A cells transfected with the four Yamanaka’s factors. B. Quantification of TRF1 transcriptional levels by q-PCR in TRF1+/+, TRF1T273A/T273A and TRF1T358A/T358A cells transfected with the four Yamanaka’s factors (iPS). Student’s t test was used for statistical analysis, p ≤ 0.05; **, p ≤ 0.01; ***, p ≤ 0.001. Error bars represent ± SE. n number of independent experiments. C. Representative Q-FISH images of metaphases spreads and quantification of the mean telomere length in TRF1+/+, TRF1T273A/T273A and TRF1T358A/T358A cells transfected either with the four Yamanaka’s factors (iPS). n, number of metaphases. Student’s t test was used for statistical analysis, *, p ≤ 0.05; **, p ≤ 0.01; ***, p ≤ 0.001. Error bars represent the SE. D. Representative image of telomeric restriction fragment (TRF) blot of TRF1+/+, TRF1T273A/T273A and TRF1T358A/T358A cell lines transfected with the four Yamanaka’s factors (iPS) at progressive passages. Numbers refer to molecular weight standards in Kb. Mean telomere length for each cell line is shown at the base of the lanes. (TIFF) [file pgen.1009410.s008.tiff]
