# Supplementary material for: AKT-dependent signaling of extracellular cues through telomeres impact on tumorigenesis
Source: PLoS Genet. 2021 Mar 9;17(3):e1009410. doi: 10.1371/journal.pgen.1009410 (PMC7942993; doi:10.1371/journal.pgen.1009410)
Supplement: Attachment — Submitted filename: Point by point response_reviewers_PGENETICS-D-20-01697.docx [file pgen.1009410.s010.docx]

**Deatiled Answwers to Reviewer #1**

Sanchez-Vazquez and colleagues provide evidence the Akt mediated phosphorylation of telomeric shelterin proteins TRF1 regulates cell growth an in vivo tumorigenesis. This report expands on previous work from this group where the Akt phosphorylation sites on mouse TRF1 were identified (Nat Commun 2017). The data are well controlled, using a range of in vitro and in vivo methods. I review a lot of papers and I must say that this manuscript was a pleasure to read-clearly written and not interpreted.

**ANSWER:** We are delighted to see that the reviewer considers that ***“the data are well controlled and that this manuscript was a pleasure to read-clearly written and not interpreted”.***

I have the following minor comments:

1. Fig. 1 the consensus sequence for Akt phosphorylation is RxRxxS/T-hydrophobic amino acid. To my eyes, the best of the 3 potential sites is the Thr273-perhaps the authors could add a few lines to refer to this in their report?

**ANSWER:** As suggested by the reviewer, we have included a sentence in the revised manuscript: “*Based on the consensus sequence for AKT phosphorylation, RxRxxS/T (Manning and Toker, 2017), out of the three identified residues T273 constitutes the most likely candidate”* (**page 6, line 113**).

2. Fig. 1E-with such small error bars, why were the experiments completed 8 times (n=8)? Usually n=3-4 is sufficient? can the authors confirm that n=8 means 8 independent experiments, and if so, how many replicates were included in each independent experiment. The new Superplot method of presenting data should be considered to allow the reader to see all date points and better interpret the data <https://rupress.org/jcb/article/219/6/e202001064/151717/SuperPlots-Communicating-reproducibility-and>)

**ANSWER:** We confirm that the data correspond to 8 independent experiments when indicated. Finally, in the new “source data file” within Supporting information, the individual measurement values corresponding to all the mutants in Fig. 1E are indicated.

3. Fid. 2D there does not appear to be a difference between the TRF1+/T273 v TRF1 T273A/T273A mutants in terms of protein levels (in contrast to TRF1T358A mutant)-can the authors comment on this please?

**ANSWER:** We respectfully disagree with the reviewer. The representative image shown in Fig. 2D (left panel) and the quantification in 5 different independent experiments show that TRF1 levels in *TRF1^+/T273A^* are higher than in *TRF1^T273A/T273A^* cells (Fig. 2D, right panel). Indeed, we also observe higher levels in *TRF1^+/T273A^* than in *TRF1^T273A/T273A^* cells when analyzing independent *TRF1^+/T273A^* and *TRF1^T273A/T273A^* clones (Supplementary Fig. 2A-B).

4. Fig. 2D, G-the authors normalise the expression level to the DMSO control-however, it is standard to normalise to the loading control (SMC1 in this case)-please revise.

**ANSWER:** We always normalize protein expression levels to its corresponding loading control, SMC1, as the reviewer points out. The data in the figures (Fig. 2D, G) represent the SMC1-normalized values relative to the levels observed in DMSO. We have changed the labeling in Y-axis for clarity purposes (*TRF1 protein levels relative to DMSO*).

5. Fig. 2F, 4, 5B, 8C-how were these images quantified and please confirm that images were blinded prior to quantification to avoid conscious and unconscious bias.

**ANSWER:** We have indicated in the Material and Methods section how the images were analyzed in each case (**page 31, line 732; page 32, line 738, line 747**). We have also indicated that image analysis was performed blindly.

6. Fig. 8 A-replot the tumor weight using a similar format used in Fig. 8G to show the proper scatter of the data.

**ANSWER:** Figure 8A represents a longitudinal time follow up of the weight of individual tumors. Five different time points are included (day 1, 6, 8, 10 and 12 post injection). Representing this data as a scatter plot, as the reviewer suggests, would imply 5 different plots (one for each time point) and would not show the weight increase with time as clear as it is now shown in Fig. 8A. The numerical data (weight of each tumor at every time point) of each tumor underlying figure 8A is included in the new “source data file” within Supporting information.

7. For discussion-many groups use pSer473 as a proxy for Akt phosphorylation; however, phosphorylation on Thr308 is also required for full activation. This should be included in future studies. Also, the use of the Akt chemical inhibitor MK22-06 is interesting as it clearly affects TRF1 phosphorylation but I am confused as to why it would also block pSer473 phosphorylation, as this site is phosphorylated by mTORC1 and DNA-PK.

**ANSWER:** MK2206 inhibits AKT autophosphorylation at both residues S473 and T308 (<https://www.selleck.eu/products/MK-2206> and references therein) and therefore fully abolishes AKT activity. We now include this in the revised manuscript text (**page 27, lines 617**).

8. For discussion-the TRF1 T344 site did not appear to be an Akt phospho site, with the T344A mutation having no affect on TRF1 levels (Fig. 1E). To me, this would have been a perfect control for any off-target/voodoo effects of CRISPR knock-in cell lines. Did the authors make the TRF1-T344A knockin cell line and show no effects on TRF1 stability/cell proliferation/tumor size etc?

**ANSWER:** We agree with the reviewer in this point. We tried several times with several different guide RNAs (n=4) and different templates (n=3) to generate the *TRF1^T344A/T344A^* cell line by CRISPR. However, none of these attempts turned out successfully. We do not know the reasons behind this failure but one potential explanation could be DNA structural constrains at this location. Given that the in vitro phosphorylation studies (Fig. 1E) showed that AKT did not phosphorylate T344, we did not continue with the generation of *TRF1^T344A/T344A^* cell line.

**Detailed Answers to Reviewer #2**

This work from the Blasco lab investigated the role of PI3K/AKT in regulating TRF1, a telomere binding protein. They identified 2 distinct targets of AKT-dependent phosphorylation (T273 & T358) in TRF1, which destabilized and perturbed the TRF1 accumulation at telomeres. The mechanism of this was linked with elevated degradation – as the reduced levels were restored by bortezimib, an inhibitor of the proteosome. The stable expression of phospho-TRF1 mutants, by knock-in of 293T cells, elicited telomere damage, accelerated telomere shortening and induced fragility suggestive of replicative complications. While the telomere shortening affect of TRF1-273 mutant cells was rescued by over-expression of wild-type TRF1, the T358 mutant did not, indicative of its dominant negative effect. However, depletion of TRF1 indicated the contrary, that the telomere shortening effect was attributed to a gain of function for TRF1. To pursue what this could be the authors tested a role for these mutants in inhibiting telomerase activity, which was not the case, as tested by ectopic expression of telomerase and through induced pluripotent stem cell generation. The authors tested whether these mutants altered the stability and binding of additional subunits of Shelterin complex. Again, an adverse affect on Shelterin, aside from TRF1 itself, was not evident.

Pursuing the metabolic link of PI3K/AKT, the authors finally examined a role for TRF1 and these phosphorylations in response to extracellular sensing of insulin and starvation. Whereas cells expressing wild-type TRF1 significantly induce the expression of TRF1 protein, cells expressing TRF1-273 or 358 did not respond. Lastly, cells expressing non-phosphorylatable TRF1 alleles did not exhibit nearly as much expansion upon xenograft transplantation. Thus, the authors conclusion is that the phosphorylation of these sites by AKT is required for the transduction of extracellular signaling to telomeres and maintain the tumorigenic proliferation of cancer cells.

The dissection of TRF1 stability and telomeric length/integrity are solid. The only suggestion is to clarify for readers the seeming contradictory statements related to the dominant negative effects. The authors state rather definitively that the phospho-mutants are not dominant negatives before changing this based on the outcomes of the shTRF1 experiments. It would be helpful to perhaps restate or rephrase this section of the text.

**ANSWER:** We thank the reviewer for the thorough review of our manuscript and greatly appreciate that the reviewer considers our data to be **“*solid*”.**

As suggested by the reviewer, we have now removed from the text the potential dominant negative effect of T358A mutation based on the lack of telomere length rescue observed upon WT-TRF1 exogenous expression (Fig. 5C). Instead, we now state that “*These findings indicate that telomere shortening phenotype induced by the TRF1^T273A/T273A^ mutant protein is the direct effect of the mutation as this effect can be rescued by over-expression of the wild-type TRF1 protein. However, telomere shortening induced by the TRF1^T358A/T358A^ mutant, is likely to be indirect as it cannot be rescued by over-expression of the wild-type TRF1 protein. It is possible that the TRF1^T358A/T358A^ mutation may also be modifying the structure of the telomere capping complex, thus impeding proper access of the wild-type TRF1 protein”* (**page16, line 342-349**). In addition, we have also removed the confusing term of dominant negative effects when describing the effect of TRF1 downregulation (Fig. 5D, Supplementary Fig. 6D) (**page 16, line 350-366**).

A minor point is that the methods do not include a description of how the IPS cells were generated and characterized, or maybe I missed this?

**ANSWER:** We have included a description of how IPs cells were generated in the revised Material and Methods section (**page 27, lines 633-635**). The IPs characterization was performed by *NANOG* expression (Supplementary Fig. 7A).

Overall, the paper is pretty comprehensive and its conclusions seem to be on point – albeit somewhat speculative. However, it should be a nice addition to the body of work on TRF1 and potential links with tumorigenesis.

**ANSWER:** We appreciate that the reviewers considers our work as **“*comprehensive with conclusions to be on point”*** and that it constitutes ***“a nice addition to the body of work on TRF1 and potential links with tumorigenesis”***.
